# Implementation of Web-Based Respondent Driven Sampling among Men Who Have Sex with Men in Sweden

**DOI:** 10.1371/journal.pone.0138599

**Published:** 2015-10-01

**Authors:** Susanne Strömdahl, Xin Lu, Linus Bengtsson, Fredrik Liljeros, Anna Thorson

**Affiliations:** 1 Department of Public Health Sciences, Karolinska Institutet, Stockholm, Sweden; 2 College of Information System and Management, National University of Defense Technology, Changsha, China; 3 Department of Sociology, Stockholm University, Stockholm, Sweden; David Geffen School of Medicine at UCLA, UNITED STATES

## Abstract

**Background:**

Respondent driven sampling (RDS) was designed to study ‘hidden’ populations, for which there are no available sampling frame. RDS has been shown to recruit far into social networks of the study population and achieve unbiased estimates when certain assumptions are fulfilled. Web-based respondent driven sampling (WebRDS) has been implemented among MSM in Vietnam and produced a sufficient sample of MSM. In order to see if WebRDS could work in a ‘hidden’ population in a high-income setting, we performed a WebRDS among MSM in Sweden to study a sensitive topic, sexual risk behaviour for HIV/STI and Internet use.

**Methods:**

A cross-sectional survey was implemented between July 11, 2012 and January 21, 2013 by using a WebRDS software. Men, fifteen years old or above, who reported having ever had sex with another man were included. The web-survey explored sociodemographics, sexual risk behaviour for HIV/STI and Internet use.

**Results:**

The WebRDS process created a sample of 123 eligible respondents. The mean age among participants was 32 years old. All respondents reported having had unprotected anal intercourse (UAI) with at least one regular and one casual sex partner during the last 12 months. On average participants reported having had UAI with three casual sexual partners and in total having had seven casual sex partners during the last 12 months.

**Conclusion:**

The WebRDS produced a sample of Internet-using MSM in Sweden who all reported sexual risk behaviour for HIV/STI during the last 12 months. It holds promise for future online studies among MSM and a possibility to reach MSM at risk for HIV/STI with interventions or information. Some challenges were found including short recruitment chains, and further research need to address how to optimize WebRDS online recruitment methods in high income settings.

## Introduction

MSM globally, as well as in Sweden, have a higher prevalence of HIV and sexually transmitted infections (STI) compared to the general population [1–3]. Therefore there is a need to study risk for HIV/STIs within MSM populations. Sexual risk behaviour for HIV/STIs among MSM includes unprotected anal intercourse (UAI) with partners of unknown HIV/STI status [4].

There are some challenges when aiming to study sexual risk behaviour among MSM. MSM populations are of unknown size and may be ‘hidden’ and ‘hard to reach‘ due to stigma [5]. A registry of sexual preference in a population is rare and in many settings such as Sweden it is prohibited by law in order to protect minority groups [6]. Thereby, it is not possible to define a sampling frame for MSM.

Studies on sexual behaviour in the general population could produce sub-samples of MSM, however such studies would require very large number of participants in order to capture a large enough sub-sample of a minority groups such as MSM. In addition, a trend of low response rates in national public health surveys in several European settings has been reported which leads to risk of bias and further difficulties in reaching a sufficient number of MSM [7–10].

Online forums for MSM, such as web pages and web-communities, are other venues where MSM can be reached for study purposes. Three online ‘banner-surveys’ have been performed among MSM in Sweden [11–13]. These studies recruited participants by web-banners on MSM web-communities with a link to the survey and were open to any MSM present online. However this convenience sampling method produces sampling bias since only MSM visiting the particular web site where the banner is shown can participate. Additionally, the vast majority of persons visiting a web site do not click on the banner/link and participate in the study and response rates have been evaluated to be around 0,1% [14].

Other available convenience sampling methods include snowball sampling, where key informants are asked to invite their MSM friends to a study [15]. Snowball sampling is successful at getting MSM to participate in studies. However, persons with many social contacts have a higher chance of being invited to the study, which introduces bias. Thereby the study population will consist of MSM with many friends, which may have implications when aiming to study sexual risk behaviour for HIV/STIs [16].

Respondent driven sampling (RDS) starts, similarly to snowball sampling, with a number of key informants within the study population who serve as the initial “seeds” [17]. After having participated in the study, the seeds are asked to distribute a certain number of invitation coupons (usually three) to their peers who also fit the study population inclusion criteria. Individuals with a valid coupon can then participate in the study and after participation they are provided coupons to distribute to their peers. The RDS recruitment chains will ideally tap far into the social network of the population under study, finally reaching individuals with few social contacts. An incentive or reimbursement for participation and successful recruitment of peers is given. This recruitment process has been shown to produce high participation rates [18]. Information about who recruits whom and the respondents’ total number of friends within the population under study (i.e. the number of peers eligible to be recruited into the study by that individual) are recorded. However, no personal identifier data needs to be recorded about the participant or their social contacts. The number of eligible peers is used to generate population estimates from the sample, weighting down persons with many contacts due to being over sampled and weighting up those with few contacts [18].

It has analytically been shown that the RDS estimations are unbiased when certain assumptions are fulfilled: (i) relationships are reciprocal, i.e., there is the same chance of A recruiting B as of B recruiting A, (ii) the network forms a single component i.e. each individual in the study population has a chance of being invited, (iii) respondents can accurately report the size of their network, (iv) sampling of peer recruitment is done with replacement, (v) each participant recruits one peer from his/her friends, and (vi) the peer recruitment is a random selection among all the participant’s friends. However, these assumptions may not be met in real life practice of RDS [18–22]. For example, RDS studies often use more than one invitation coupon. Sampling is usually conducted without replacement and each respondent is only allowed to participate once. Respondents who receive a coupon may be more likely to distribute it to close friends rather than randomly among all friends within the network, particularly if the study touches upon a sensitive topic such as sexual behaviour [22–24].

Implementation of the RDS method online could achieve the same benefits as in real life. In addition the online setting offers privacy and the participant can choose when and where to participate. Finally, by recruiting via email, WebRDS may be able to reach MSM who are not active on MSM web-communities and pages.

WebRDS has been implemented among MSM in Vietnam to study sexual risk behaviour for HIV/STIs and it produced a sufficient sample of Internet-using MSM [25]. In order to see if the WebRDS recruitment method could work in a ‘hidden’ population to study a sensitive topic in a high-income setting, we performed a WebRDS among Internet-using MSM in Sweden to study sexual risk behaviour for HIV/STI and Internet use.

## Methods

### Study design and population

An online cross-sectional survey was performed applying a WebRDS software for recruitment, participation and incentives for participation and recruitment during July 11, 2012 to January 21, 2013. Eligibility criteria included being a man, 15 years old or older, reporting having had any type of sex (including oral sex or fondling) with another man. The survey was in Swedish. Participants were required to have an email address to be able to recruit peers.

### Web-survey design

The design of the web page aimed at being easy to navigate and to give a professional impression. Information regarding the study’s aim, methods, and investigators was provided.

The web-survey included modules on: sociodemographic information, social networks, sexual risk behaviour for HIV/STI, Internet use including email and MSM web-communities and -pages. The web-survey used skip patterns and participants answered a minimum of 25 questions and a maximum of 49 questions.

Data on participants’ network size was gathered by asking each participant how many study eligible contacts could they invite through the WebRDS recruitment method if there were no limitation [17].

The web-survey and WebRDS recruitment system were piloted with ten key informants representing different age groups (15–25, 25–35, 35–55, >55 years old) and sexual orientations (homo-, bi- sexual and transgender). Key informants evaluated their experience of usage and interviews were performed with focus on user-friendliness of the web-features and to ensure appropriateness of questions. The time it took to participate was recorded to establish that online participation was not too time consuming. The formative phase of the study was conducted with assistance from the non-governmental organization, the Swedish Federation for Lesbian, Gay, Bisexual, Transgender and Intergender (LGBTI) Rights in Stockholm.

### WebRDS sampling and recruitment

Recruitment was performed through WebRDS using a software previously described [25]. The software included features for email recruitment, participation in the web-survey and sent incentives for participation and recruitment of peers via email [25]. Initially, 13 seeds were recruited and initiated the study. Four (4) seeds were identified through collaboration with the Swedish Federation for LGBTI Rights and nine seeds (9) through online recruitment of MSM members of Scandinavia’s largest web-community for LGBTI (Qruiser) [26].

Seed participants were selected to reflect the diversity of MSM living in Sweden by taking into account age (age groups 15–25, 25–35, 35–55, >55 years old), sexual orientation (homo-, bi-sexual, transgender) and place of residence (urban or rural county).

The recruitment of seeds through the web-community was performed by sending an invitation message to the most recent member who logged in on July 11, 2012 per county of residence, reporting being male and looking for men. The nine invited that responded positively became seeds. They represented different age group and reported living in different counties. The seeds were informed regarding the study and the function of them as seed participants. Seeds were invited to participate via email or message in their web-community inbox including a unique link. The link took them to a web-page where the study information was repeated and they could give their consent to participate by an active click that took them to the web-survey.

After answering the web-survey, participants were invited to recruit via email up to four MSM friends to participate. Participants were asked to enter their email address and recruitment emails with four unique links to the web-survey were sent to this email address. Seeds could forward these invitation emails to friends or copy and send through the media of their preference (e.g. Web-community, chat, Facebook).

The WebRDS was implemented online between July 11, 2012 and January 21, 2013 with a break due to a server failure between August 23 and September 20, 2012. Individuals in the respective recruitment chains affected by the server failure were informed when they tried to reach the survey page. The last participants in each recruitment chain were sent an information email regarding the server failure and study break. When the server was functioning again, one month later, new invitation emails were sent informing the last person that participated in the recruitment chain that it was possible to recruit and participate once again. An additional 16 seeds were invited on September 20, 2012 and another eight seeds on November 2, 2012. The same recruitment process on the web-community as described above was used. The additional seeds represented a diversity of age groups and counties in Sweden.

### Incentive for participation and recruitment

Participants were offered an incentive to participate and recruit friends to the study. The recommended value of an incentive/reimbursement for RDS in a high-income setting is about 20USD [27]. However, in Sweden the cut off for when a gift is considered tax-free is 12USD and incentives had to be kept under this value [28]. The incentive used was a gift certificate for one month of the highest standard of membership at the same Web-community used for recruitment of some seeds (monetary value of approximately 4 USD). Participants were given one gift certificate for participation in the web-survey and additional gift certificates for every person they recruited who participated. The incentive was piloted among key informants to suit preference of different parts of the MSM study population.

### Data Cleaning

Data entry was performed by the participants through the WebRDS software program for a total of 148 participants. Seeds were included in the sample [29]. Double participation was identified through duplication of email addresses and 18 participants were removed. Participants were further excluded due to that they did not fit the inclusion criteria, i.e. 4 due to reporting being a woman and 2 due to reporting never having had sex with a man. Respondents who took less than 3 minutes to answer the questionnaire were excluded due to that it was deemed infeasible to read and answer the questionnaire in such short time frame, 1 participant were removed for this reason. A total sample of 123 participants remained.

### Analysis

Descriptive analysis was performed using STATA 12.0 software. RDS estimates were calculated using the RDSII estimator and a design effect of 2 [21, 30]. The individual’s ego-network size was estimated by asking participants the question: ‘How many MSM who are 15 years or older, would you like to invite to this study by the described online recruitment process, if possible to invite more than four?’

### Ethics

Ethical approval was obtained from the Regional Ethical Review Board in Stockholm, Sweden. Informed consent by participants was asked for and provided electronically, providing the same possibility of opting in or out, as in writing, but instead, after having read the study information, participants were asked to actively click to confirm ‘Yes, I want to participate in the study’. The WebRDS software program saved the participants informed consent information. No personal identification data such as name or personal ID number was gathered, and the data collected was hence anonymous to everyone including the research team. The study targeted sexual behaviour and the age of 15 years old was set as the lower limit of age for participants. Fifteen years old is the age when consenting individuals may engage in sexual acts according to Swedish law [31]. Hence no parental or guardian consent for participants between 15–18 years old was obtained and this procedure was approved by the Regional Ethical Review Board in Stockholm. All pages included a log out button, which removed the survey from the computer used and took the user to an online search engine. Participants were given instructions on how to delete browser history. Login links to the survey were unique and could not be accessed from more than one computer simultaneously. Communication between the survey webpage and the server was encrypted.

## Results

### Characteristics of the MSM participants in the WebRDS sample

The mean age among participants was 32 years, with a range from 19 to 73. 28% of the respondents had university-level education and about half of the respondents (51%) had finalized secondary school (see Table 1).

**Table 1:** Characteristics of participants.

Variable	Covariate	Observation/Missing	Mean /proportion	95% CI	RDS estimate	95% CI
Age		121/2	32.06	[29.88 34.24]	33.27	[30.19 36.36]
Nationality	Swedish	121/2	0.86	[0.80 0.92]	0.81	[0.72 0.90]
County of residence	Stockholm county	121/2	0.25	[0.17 0.32]	0.22	[0.11 0.33]
Education	Tertiary	121/2	0.28	[0.20 0.36]	0.27	[0.15 0.38]
	Secondary		0.51	[0.42 0.60]	0.44	[0.32 0.57]
	Vocational training		0.12	[0.07 0.18]	0.11	[0.03 0.19]
	Primary (grade 1–9)		0.08	[0.0 0.14]	0.19	[0.00 0.27]
Occupation	Employed including students and retired respondents	121/2	0.79	[0.71 0.86]	0.82	[0.72 0.93]
Sexual orientation	Homosexual	121/2	0.75	[0.68 0.83]	0.78	[0.67 0.89]
	Bisexual		0.23	[0.16 0.31]	0.29	[0.18 0.40]
	Heterosexual		0.03	[0.00 0.06]	0.02	[0.00 0.07]
	Asexual		0.02	[0 0.04]	0.00	[0 0.04]
	Don’t know		0.03	[0 0.06]	0.02	[0 0.06]
Network size	Reported number of MSM possible to invite	105/18	31.03	[11.49 50.57]		
Web-community profile	1 profile	116/7	0.79	[0.72 0.87]	0.81	[0.71 0.91]
	2 profiles		0.09	[0.04 0.15]	0.09	[0.01 0.16]
	≥3 profiles		0.11	[0.05 0.17]	0.10	[0.02 0.18]
Level of Web-community membership	Highest level	116/7	0.38	[0.29 0.47]	0.33	[0.21 0.46]

**Fig 1:**
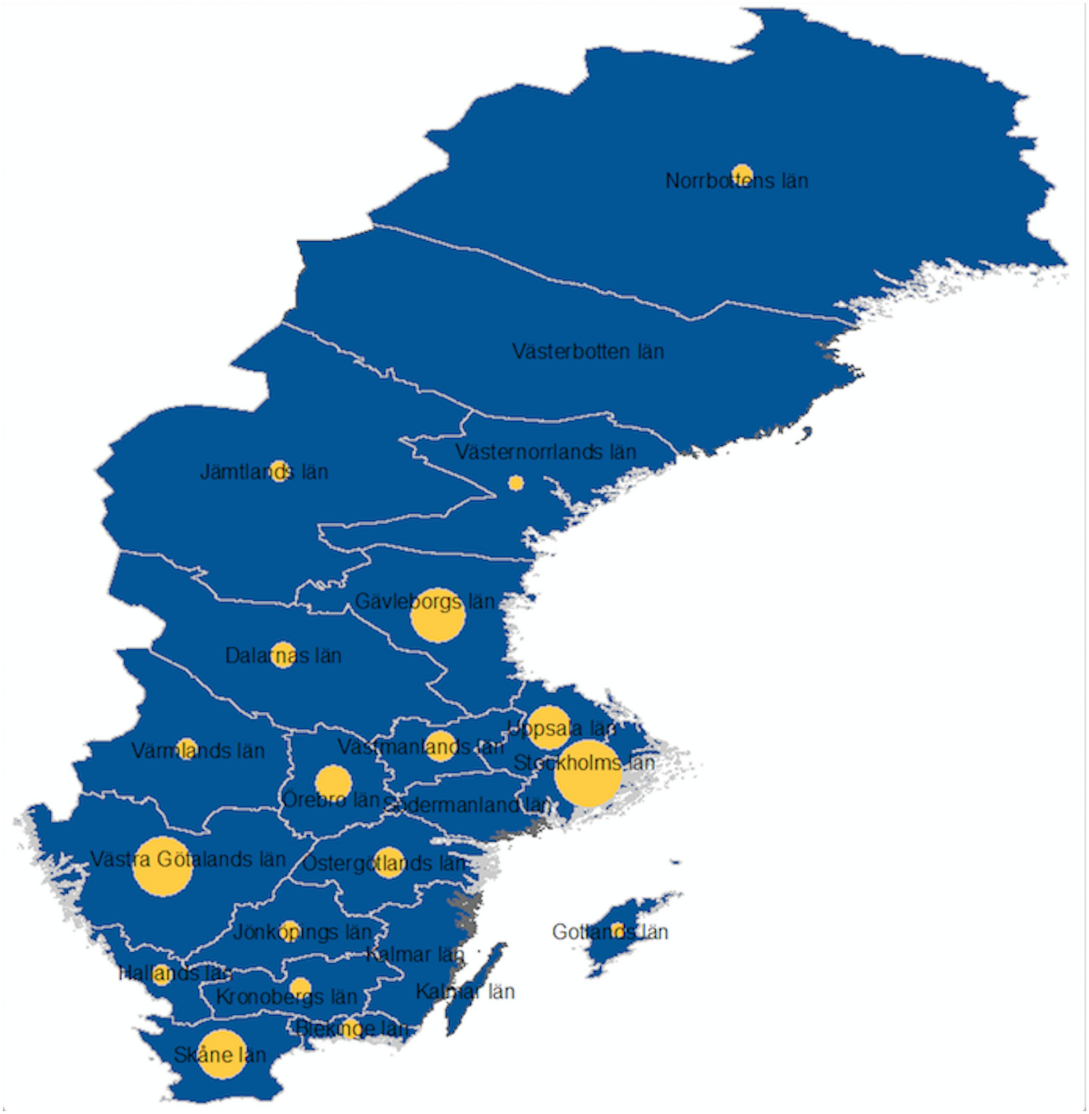
MSM study participation per county in Sweden.

Participants came from most counties in Sweden, including rural areas with low population density (Fig 1). 25% reported residing in Stockholm County, which is the home to about 20% of Sweden’s population [32]. The majority reported being homosexual and about a fifth reported being bisexual.

### WebRDS recruitment performance

**Fig 2:**
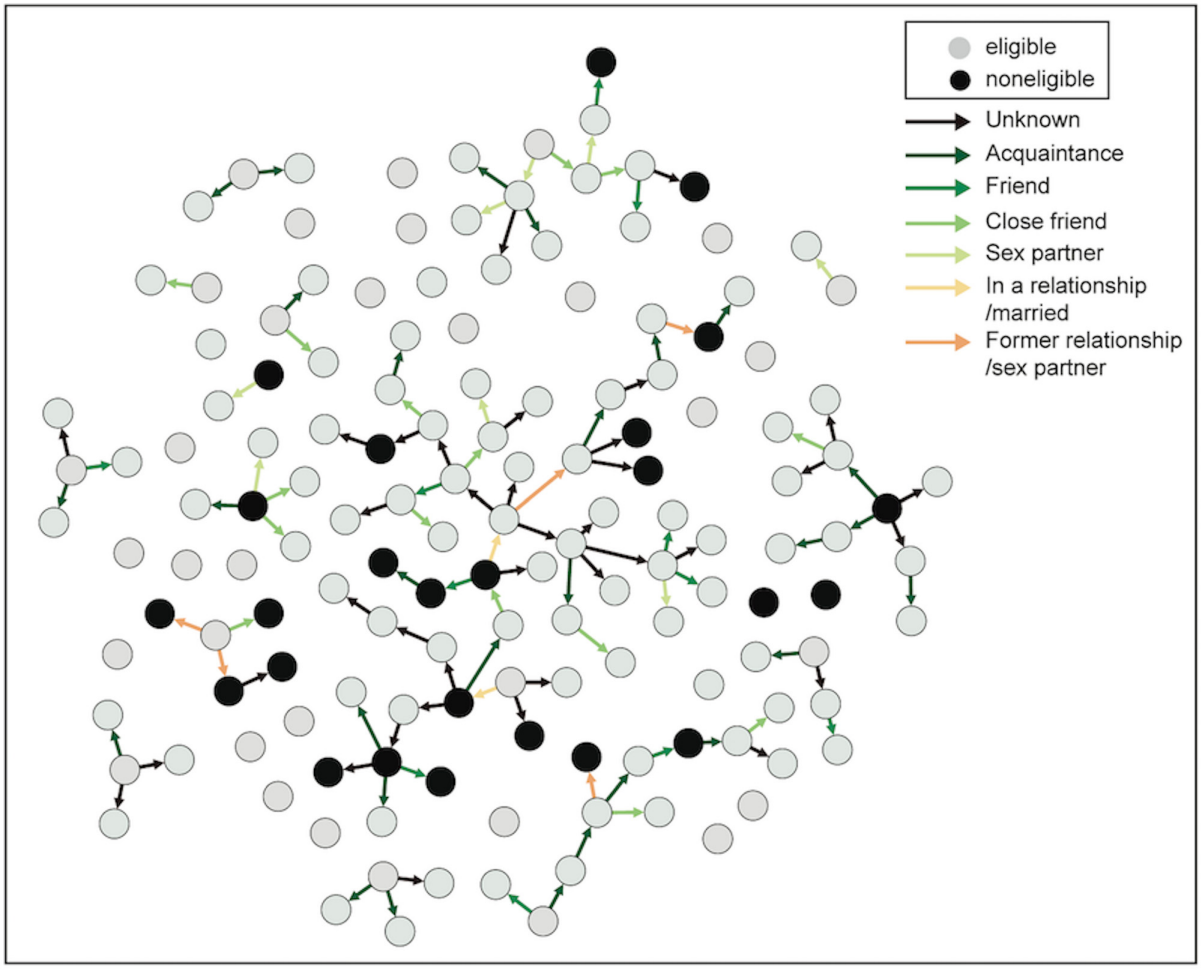
WebRDS recruitment chains for 148 MSM participants.

RDS recruitment chains varied in length between 1–9 waves. Three recruitment chains reached more than three waves (Fig 2). The final sample forms a network of 17 recruitment chains with at least two respondents on the chain and 41 disconnected respondents isolated due to exclusion of their links according to exclusion criteria or not recruiting additional respondents.

**Fig 3:**
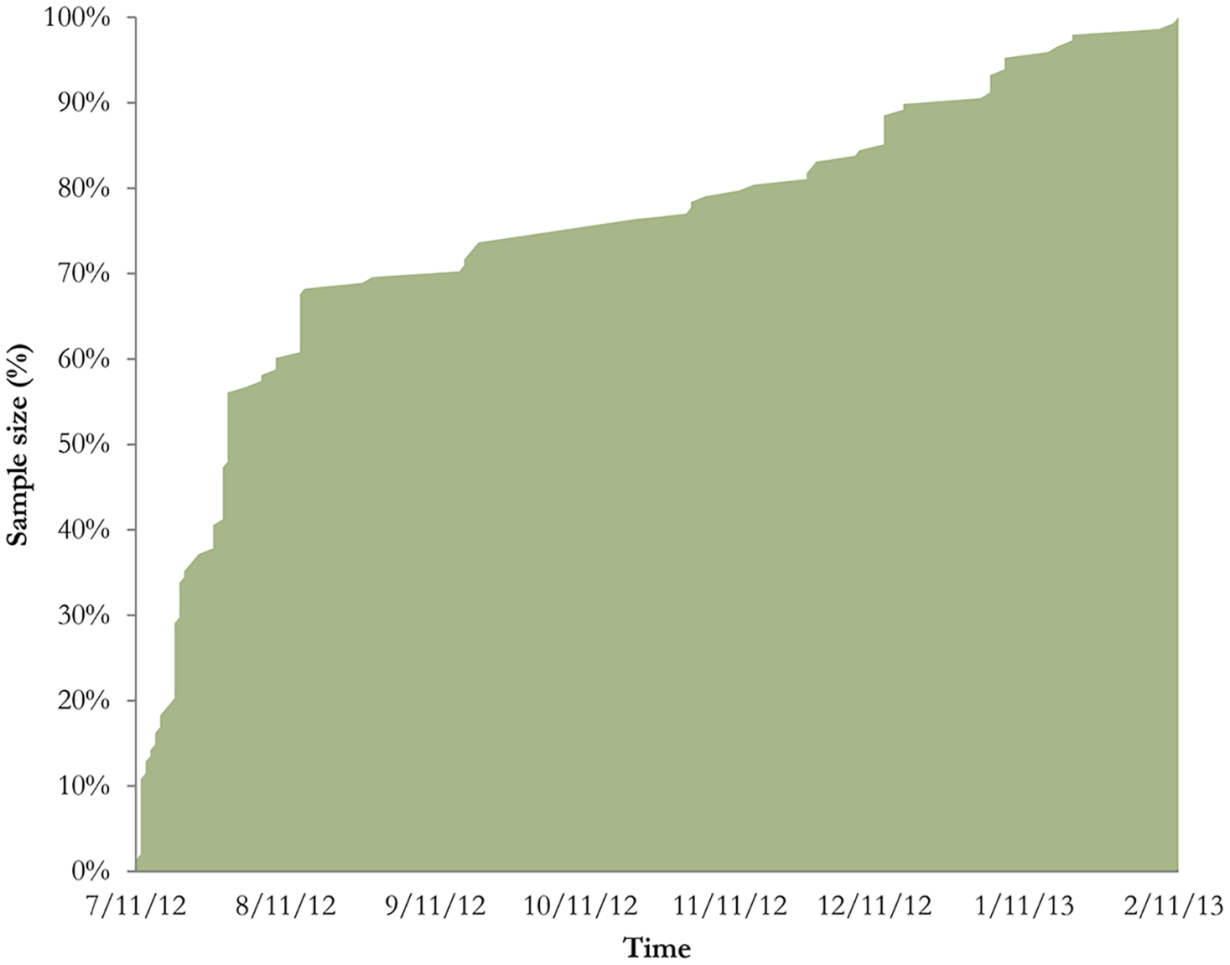
Participation in the WebRDS over time.

Two seeds and five participants were successful at recruiting four respondents, the maximum number possible. Seeds represented 33 out of 123 eligible participants i.e. 30% of the final sample. About two thirds (68%) of the final sample was recruited before the server failure on August 23, 2012. Ten (out of 13) recruitment chains did not start again after being re-invited when the server was functioning again after the breakdown. The three recruitment chains that did start recruiting after the server breakdown, contributed with an addition of six more respondents. Recruitment chains started after the server failure were slower in comparison to before the server failure, as shown in Fig 3.

To reach stability of sampling waves i.e. equilibrium, more recruitment waves would have been needed. We compared the WebRDS sample with and without the last 40 respondents to analyse if the sample composition was still changing. A change in proportion of 0.02 of MSM living in Stockholm, 0.03 having a university education, 0.025 being unemployed, and 0.002 in having a gold membership was found. Numeric change for age was 2.5 and for average personal network size 0.23.

### Sexual risk behaviour for HIV/STI

**Fig 4:**
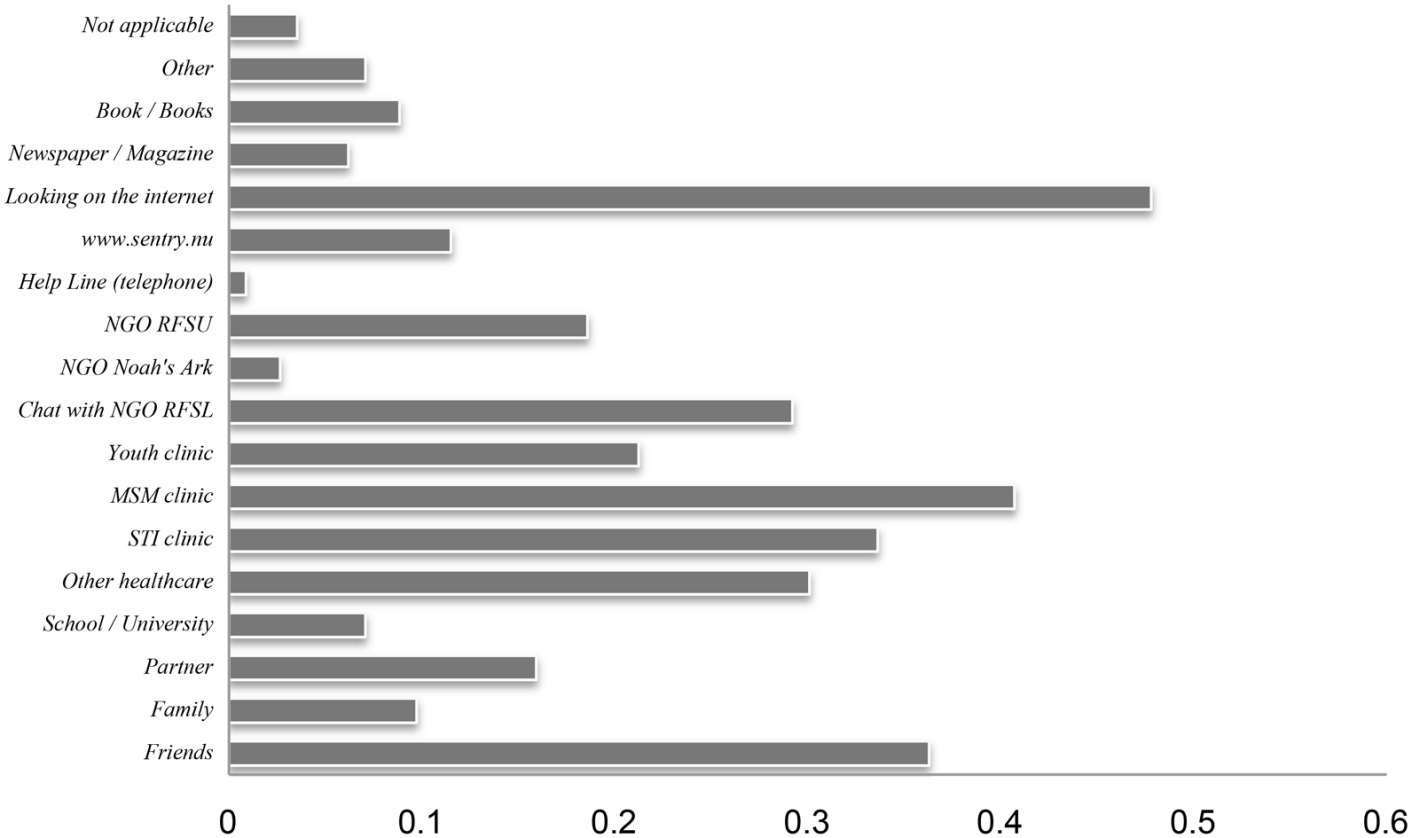
HIV/STI prevention resources usage among MSM respondents.

All respondents reported having had UAI with at least one regular and at least one casual sex partner during the last 12 months (see table 2). On average MSM respondents reported 4.2 regular male sex partners during the last 12 months of which they reported UAI with on average 2.9. Average of casual male sex partners in the last 12 months were 7.2 of which they reported UAI with on average 3.0. Half of MSM respondents reported that when in need of HIV/STI information and prevention services they would search online, indicating a preference for Internet-based resources (Fig 4).

**Table 2:** Male sexual partner frequency during the last 12 months.

Variable	Observation/Missing	Sample mean /Proportion	95% CI	RDS estimate	95% CI
Number of regular male sexual partners	121/2	4.27	[2.40 6.15]	4.89	[2.24 7.54]
Number of regular male sexual partners for unprotected anal intercourse	121/2	2.88	[1.33 4.44]	3.29	[1.09 5.49]
Number of casual male sexual partners	121/2	7.17	[5.08 9.25]	6.76	[3.81 9.71]
Number of casual male sexual partners for unprotected anal intercourse	121/2	2.96	[1.28 4.64]	2.81	[0.44 5.19]

### Internet usage

**Fig 5:**
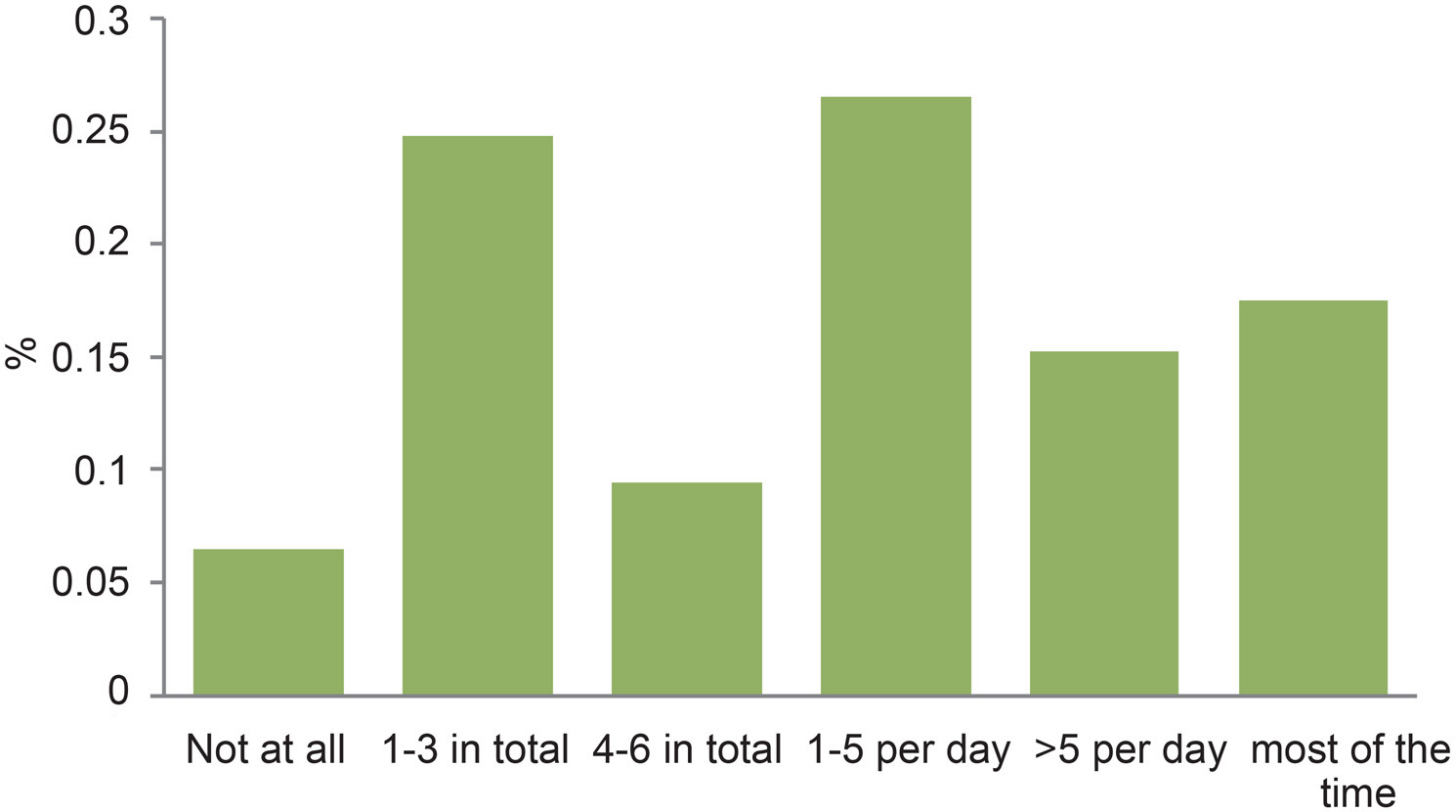
Email Usage per week among MSM respondents.

**Fig 6:**
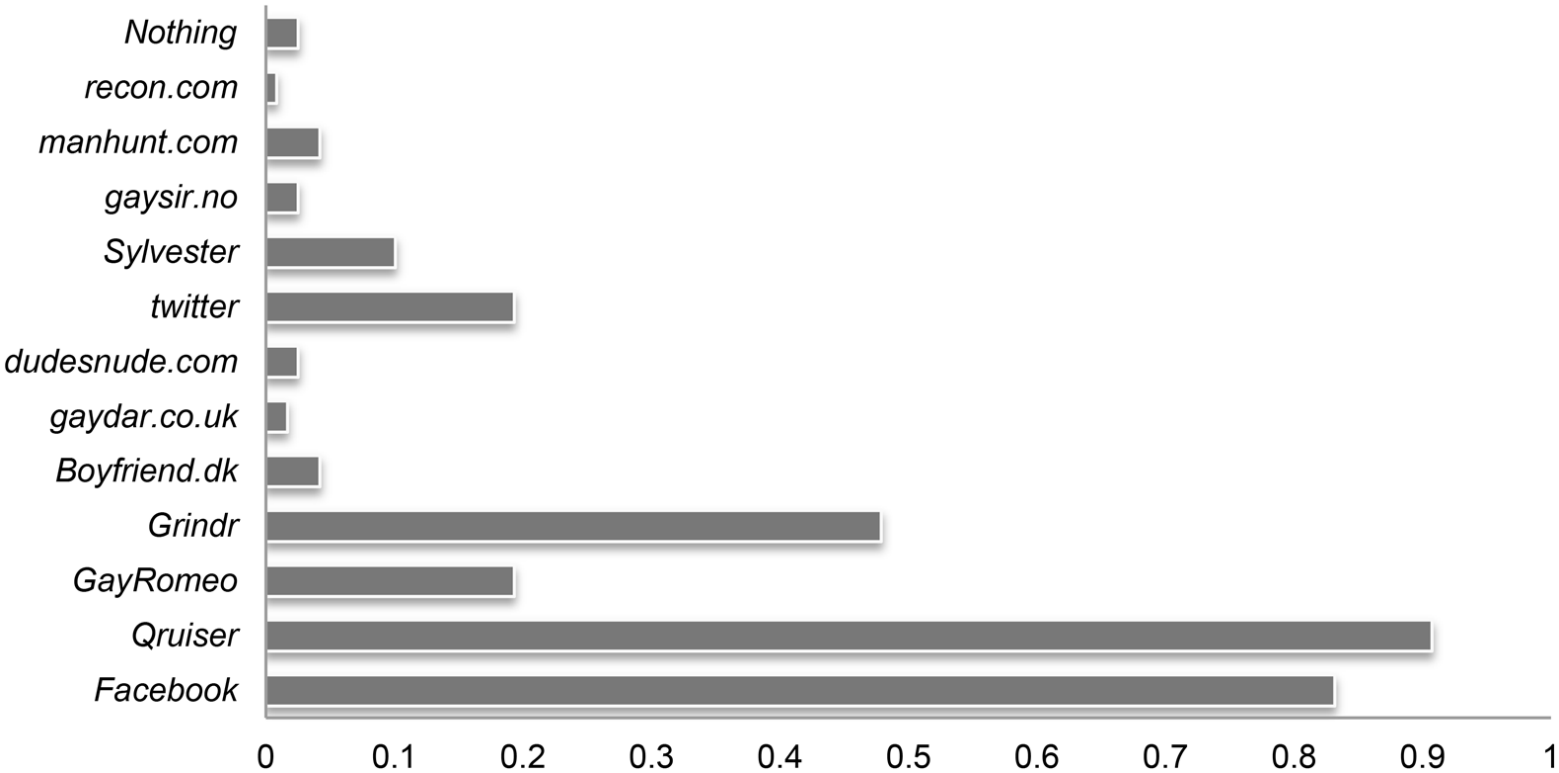
Use of online social networks among MSM respondents.

The majority (60%) of participants checked their email once or more per day (Fig 5). A variety of Internet-based social networks were used, indicating diverse online presence among MSM. The most commonly used was Scandinavia largest web-community for LGBTI (Qruiser) (Fig 6) (26). Web-community membership was reported by 94% (116/123) of participants.

## Discussion

The WebRDS recruitment method was able to recruit a sample of Internet-using MSM living in a wide variety of counties in Sweden. In addition, it was possible to include sensitive questions on sexual behavior in the WebRDS survey. All of the participants reported having engaged in UAI with a casual sex partner over the last 12 months indicating risk behavior for HIV/STI. The sample size achieved was small with short recruitment chains, which calls for additional strategies in implementation of future WebRDS in this setting.

The RDS-adjusted estimates have been shown to be unbiased when the six RDS assumption are fulfilled [21]. However, these assumptions are commonly not met in empirical RDS studies [29]. In this study, the majority (65%) of study participants reported reciprocal relationships indicating some (35%) violation to the reciprocal assumption (criteria i). The pilot interviews reported that the MSM population was well connected online through web-communities. However some parts of the transgender population might not be a part of this MSM network due to not identifying as male or gay (criteria ii). Study participants reported their network size after having participated themselves in order to be aware of what inviting friends included in detail. The aim was to enable participants to report their network size accurately (criteria iii). This study fulfilled the assumption of allowing sampling with replacement i.e. the same person was allowed to participate and recruit more than once (criteria iv), which is usually not allowed in real life RDS [29]. Each participant was allowed to recruit more than one peer (criteria v), which is common in RDS, (4 was allowed). The participants reported a variety of relationships with the person who recruited them to the study representing diversity. However, like in any RDS, we cannot establish that random recruitment was achieved (criteria vi). In summary, with certainty criteria number i and v was violated and criteria number iv was fulfilled. Regarding the remaining criteria the study aimed to fulfill criteria ii, iii and vi.

Interestingly, noting these limitations of the WebRDS performance and small sample recruited, the RDS-adjusted estimates are similar to the reported data i.e. when treated as a convenience sample. No RDS-estimates differs widely from the reported data, indicating that for these particular variables in this particular population there is not a strong correlation to individuals reported network size.

The WebRDS recruitment process died earlier than expected and produced rather short recruitment chains. Equilibrium could hence not be established. The technical challenges with a server break down affected the recruitment chains. Other possible reasons could be that peer recruitment was only available via email. Further, the web-survey was not adapted to tablets or smart phone format, which could have enabled easier participation and peer recruitment. Two other WebRDS studies using email recruitment among the general population in the Netherlands and Thailand reports on similar trends with short recruitment chains and large number of seeds [33, 34].

The unsuccessful recruitment chains reported can possibly be due to that the incentive/reimbursement for participation and recruitment might not have been attractive enough for the majority of the study population. To have an attractive incentive was found to be a key factor for successful recruitment in a WebRDS study among MSM in Vietnam[25]. An attractive incentive to participate in a high-income country such as Sweden has been evaluated to an economical value of above 20 USD [27]. This is also the cut off for when a gift is considered tax-free in Sweden [28]. Therefore this study deemed it not feasible to have incentives surpassing this value. A more attractive incentive could have increased participation, recruitment wave length and the sample size. However, Sweden is a high income setting where economical incentives may hold some attraction to weaker economical groups such as youth, students or unemployed but possibly not to all in the MSM population. Other features to ease participation such as easy access and invitation processes are probably equally important as higher incentives.

MSM respondents had on average had UAI with 3 casual sex partners during the last 12 months. As receptive UAI is estimated to hold a 1.4% per act probability of HIV transmission even few casual sex partners for UAI holds a transmission risk [4]. MSM is the group with the highest incidence of domestic HIV transmission in Sweden [3]. WebRDS is a possible method for reaching MSM that engage in sexual risk behaviour for HIV/STI. In addition, WebRDS may hold potential for delivering interventions targeting MSM engaging in sexual risk behaviour for HIV/STI, such as information and behavioural change interventions aiming to increase safe sex.

The majority of the sample checked their email once or more than once a day, indicating that an invitation email would be seen within a short time frame. However an invitation email to a study might not be prioritized to answer to when scanning the email inbox [35]. In addition, other Internet-based invitation options than email should be explored. Which Internet forum that is suitable for WebRDS recruitment is dependent on the Internet-using population under study and may vary substantially between different settings. In addition, it may be valuable to include several different ‘easy to use’ invitation options online to attract different sorts of Internet users, such as web-communities, chat, Facebook, and MSM apps such as Grindr or Scruff.

In WebRDS it is important to consider that seeds have Internet-based social networks particularly regarding the web-based approach (email, chat, MSM Web-community) that will be used for WebRDS recruitment. We recommend performing formative research regarding preferred web-based approaches for recruitment among the study target population before designing the WebRDS recruitment software.

When studying sexual behaviour there is a risk of bias in self-response surveys. Response desirability bias may have been reduced by the privacy of the online setting, peer recruitment process and by that no personal identifier data was collected. To diminish recall bias we asked about sex partners within the time frame of the last twelve months.

In conclusion, WebRDS produced a sample of Internet-using MSM in Sweden who all reported sexual risk behaviour for HIV/STI during the last 12 months. WebRDS holds promise for future online studies among MSM and a possibility to reach MSM at risk for HIV/STI with interventions or information. Some challenges were found including short recruitment chains, and further research need to address how to optimize WebRDS online recruitment methods in high income settings.
